# Punicalagin protects H9c2 cardiomyocytes from doxorubicin-induced toxicity through activation of Nrf2/HO-1 signaling

**DOI:** 10.1042/BSR20190229

**Published:** 2019-05-14

**Authors:** Mingfang Ye, Linlin Zhang, Yuanming Yan, Huizhong Lin

**Affiliations:** Department of Cardiology, Union Hospital, Fujian Medical University, No. 29 Xinquan Road, Fuzhou City, Fujian Province 350001, P. R. China

**Keywords:** cardiotoxicity, doxorubicin, mitochondrial dysfunction, Nrf2/HO-1 signaling, punicalagin

## Abstract

Doxorubicin (DOX) is a wide-spectrum antitumor agent, but its clinical application is largely limited by its cardiotoxicity. Therefore, identification of effective agents against DOX-induced cardiotoxicity is of critical importance. The present study aimed to determine the beneficial role of punicalagin (PUN), a polyphenol isolated from pomegranate, in DOX-induced cardiotoxicity *in vitro* and explored the underlying mechanisms. H9c2 cardiomyocytes were pretreated with different concentrations (50, 100 and 200 μM) of PUN prior to DOX exposure. The results showed that PUN pretreatment significantly increased cell viability, inhibited lactate dehydrogenase (LDH) release and suppressed cell apoptosis induced by DOX. Additionally, PUN pretreatment attenuated the loss of mitochondrial membrane potential and cytochrome c release. Besides, PUN further enhanced the expression of nuclear Nrf2 and HO-1 in DOX-treated H9c2 cells, and the aforementioned beneficial effects of PUN were partially abolished by small interfering RNA (siRNA)-mediated Nrf2 knockdown. Hence, our findings clearly revealed that PUN might be a promising agent for alleviating the cardiotoxicity of DOX, and Nrf2/HO-1 signaling might serve a critical role during this process.

## Introduction

Doxorubicin (DOX), an anthracycline antibiotic, is one of the most effective chemotherapeutic agents widely used in the management of a wide spectrum of human neoplasms [1]. But its clinical use is largely hampered due to its toxicity to various human organs, especially to heart [2]. About 10% of patients who received DOX or its derivatives will develop cardiac complications up to 10 years after the cessation of chemotherapy [3]. Hence, it is of great necessity to identify effective therapeutic agents against DOX-induced cardiotoxicity.

**Figure 1:**
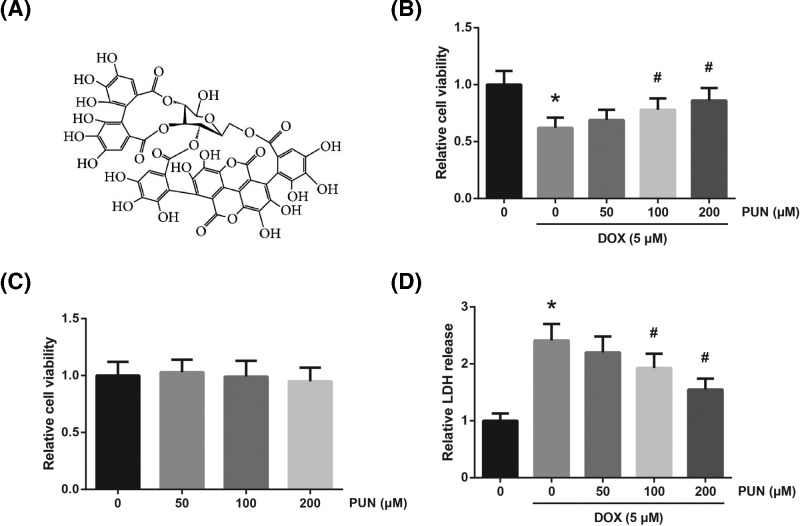
PUN protects against DOX-induced H9c2 cell death (**A**) Chemical structure of PUN. (**B**) H9c2 cells were pretreatment with different concentrations of PUN for 2 h prior to DOX stimulation, and the cell viability was detected by MTT assay. (**C**) Cytotoxicity of different concentrations of PUN in H9c2 cells under normoxic condition was detected by MTT assay. (**D**) The cell injury of H9c2 cells was assessed by measuring the amount of lactate dehydrogenase (LDH) release. The results are expressed as mean ± S.D. (standard deviation)). ^*^P<0.05 vs control cells; ^#^*P*<0.05 vs DOX-treated cells.

Natural sources are very promising materials for the identification of novel biologically active compounds. Punicalagin (2,3-hexahydroxydiphenoyl-gallagyl-D-glucose; PUN; Figure 1A), the main ellagitannin polyphenol derived from pomegranate peel or seeds, exhibits a wide range of pharmacological properties. For example, Chen et al. [4] indicated that PUN reduced oxidative stress and stimulus-induced apoptosis in human placental trophoblasts, and Ding et al. [5] reported that PUN protected against ischemia/reperfusion-induced myocardial injury by suppressing cardiomyocyte apoptosis. The main goal of the present study was to investigate the potential beneficial role of PUN against DOX-induced cardiotoxicity using *in vitro* cellular model and to elucidate the underlying molecular mechanisms.

## Materials and methods

### Cell culture and treatments

Embryonic ventricular rat heart derived H9c2 cardiomyoblasts, purchased from the Cell Bank of Chinese Academy of Sciences (Shanghai, China), were cultured in Dulbecco’s Modified Eagle’s Medium (DMEM; Invitrogen, Carlsbad, CA, U.S.A.) containing 10% fetal bovine serum (FBS; Invitrogen), 100 U/ml of penicillin and 100 U/ml of streptomycin at 37°C in a humidified atmosphere of 5% CO_2_.

The H9c2 cells were plated in 96-well plates at a density of 3 × 10^3^ cells/well and challenged with 5 μM DOX (HPLC > 98%; Sigma–Aldrich, St. Louis, MO, U.S.A.) for 24 h. 2 h prior to DOX stimulation, the cells were randomly pretreated with 50, 100 and 200 μM PUN (HPLC > 97%; Tauto Biotech, Shanghai, China) with DMSO as a vehicle, respectively.

Small interfering RNA (siRNA) targeting rat Nrf2 (si-Nrf2) was obtained from GenePharma (Shanghai, China). The non-specific siRNA (si-NC) consisted of a non-targeting used as a control. H9c2 cells were seeded at 5 × 10^4^ cells/well in 6-well plates to achieve 80–90% confluence, and then transfected with si-Nrf2 or si-NC at 10  nM using a Lipofectamine 2000 kit (Invitrogen), and after 48 h, the transfection efficacy was confirmed by Western blot analysis.

### MTT assay

Cell viability was assessed by 3-(4,5-Dimethylthiazol-2-yl)-2,5-diphenyl tetrazolium bromide (MTT) assay. In brief, after the aforementioned treatments, the cells were incubated with 10 μl of MTT solution (5 mg/ml; Roche Diagnostics, Basel, Switzerland) for additional 4 h. The supernatants were then removed, and the formazan crystals were dissolved in 150 μl DMSO. The absorbance value was detected at 570 nm using a microplate reader (MultiskanEX, Lab systems, Helsinki, Finland).

### Lactate dehydrogenase release assay

Cell injury was evaluated by measuring the lactate dehydrogenase (LDH) leakage. The released LDH in the collected culture medium was determined by spectrophotometry using the CytoTox 96 non-Radioactive Cytotoxicity Assay kit (Promega, Madison, WI, U.S.A.).

### Cell apoptosis analysis

Cellular apoptosis was detected using the Annexin V-FITC/PI kit (Beyotime, Shanghai, China). In brief, the cells were harvested after experimental treatments, washed twice with cold PBS, and then labeled with 10 μl Annexin V-FITC and 5 μl PI in the dark for 10 min at room temperature. Then the cells were subjected to FACScan flow cytometer (Becton-Dickinson, San Jose, CA, U.S.A.) equipped with Cell Quest software.

### Measurement of intracellular ROS level

Intracellular ROS generation was determined using the membrane-permeable fluorescent probe 2′7′-dichlorodihydrofluorescein diacetate (DCFH-DA; Cell Biolabs Inc., San Diego, CA, U.S.A.). Briefly, following different treatments, the cells were washed twice with PBS and incubated with 5 μM DCFH-DA at 37°C for 20 min in the dark. The DCFH-DA fluorescence was then detected by FACScan flow cytometer.

### Measurement of mitochondrial membrane potential

The mitochondrial membrane potential (MMP) was measured by a JC-1 assay kit (Beyotime). After the treatments, the cells were washed with PBS and stained with 5 µM JC-1-fluorescent dye. After incubation at 37°C for 20 min, the cells were washed with PBS and analyzed using a microplate reader. The results were expressed as the ratio of red (JC-1 aggregates) to green (JC-1 monomers) fluorescence.

### Western blot analysis

Total cellular protein was extracted using a total protein extraction kit (KeyGen Biotech, Nanjing, China), and the lysates of cytoplasm and mitochondria were prepared using the Mitochondria/Cytosol Fractionation kit (Abcam, Cambridge, MA, U.S.A.). The cytoplasmic and nuclear protein extracts were prepared using a nuclear and cytoplasm protein extraction kit (KeyGen Biotech). Equal amounts of denatured protein samples were separated by SDS/PAGE, and then transferred to PVDF membranes (Millipore, Billerica, MA, U.S.A.). Following blocking in 5% fat-free milk for 1 h, the membranes were incubated with the appropriate primary antibodies overnight at 4°C, followed by incubation with the secondary antibody at room temperature for 1 h. The signals were visualized using an enhanced chemiluminescent detection kit (Beyotime). The results were normalized to GAPDH, COX IV and Histone H1.

### Statistical analysis

All experiments were repeated at least three times. The experimental data are presented as the mean ± standard deviation (S.D.). The significance of differences between groups was determined by Student’s *t*-test or one-way ANOVA followed by Student–Newman–Keuls test using GraphPad Prism version 6.0 (GraphPad Software, Inc., San Diego, CA, U.S.A.). All *P*-values were two-sided, and *P*<0.05 was considered statistically significant.

## Results

### PUN protects against DOX-induced H9c2 cell death

First, as indicated by MTT assay, the viability of H9c2 cells was notably decreased after 24 h of DOX stimulation, and pretreatment with 100 and 200 μM PUN markedly rescued the impaired cell viability (Figure 1B). Besides, as shown in Figure 1C, different concentrations of PUN exerted no obvious toxic effects on H9c2 cells under normal condition. Additionally, DOX treatment enhanced the release of LDH in H9c2 cells, which could be concentration-dependently suppressed by PUN (Figure 1D).

### PUN inhibits H9c2 cells from DOX-induced apoptosis

**Figure 2:**
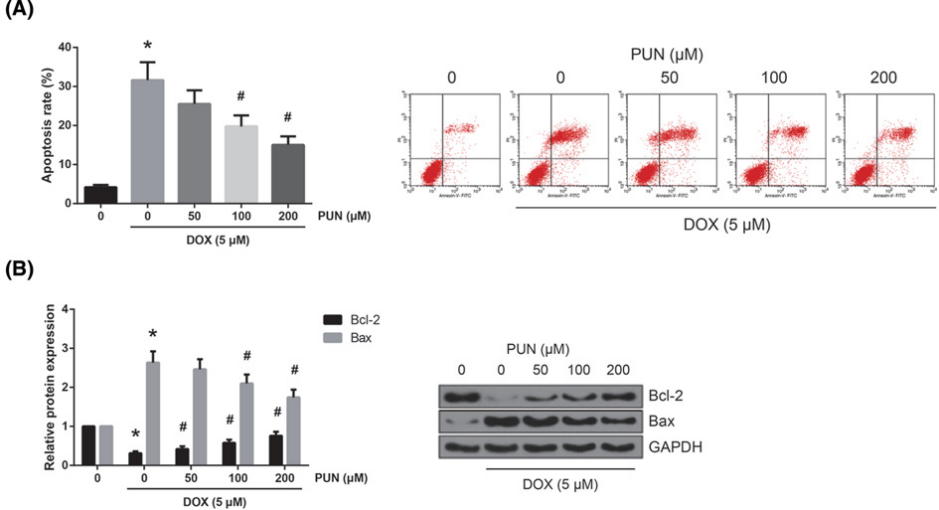
PUN inhibits H9c2 cells from DOX-induced apoptosis (**A**) The apoptotic rates of H9c2 cells were measured by flow cytometry following Annexin-V/PI staining. (**B**) Western blot analysis of Bax and Bcl-2 protein expression in H9c2 cells. The results are expressed as mean ± S.D. *P<0.05 vs control cells; ^#^*P*<0.05 vs DOX-treated cells.

Cellular apoptosis was assessed by Annexin V/PI double staining, and the results showed that the number of apoptotic cells was notably increased upon DOX stimulation, and PUN concentration-dependently reduced the apoptotic ratio (Figure 2A). The Bax/Bcl-2 ratio determines the apoptotic potential of a cell, and here we found that upon DOX stimulation, the expression of Bcl-2 was markedly reduced, whereas the expression of Bax was evidently increased, and these effects were concentration-dependently attenuated by PUN pretreatment (Figure 2B).

### PUN reduces intracellular ROS accumulation in DOX-treated H9c2 cells

**Figure 3:**
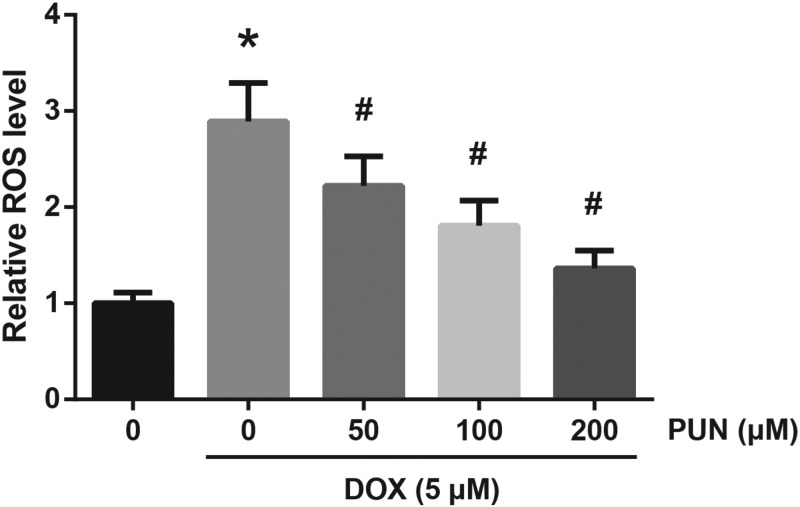
PUN reduces intracellular ROS accumulation in DOX-treated H9c2 cells The accumulation of ROS in H9c2 cells was measured by flow cytometry following DCFH-DA staining. The results are expressed as mean ± S.D. *P<0.05 vs control cells; ^#^*P*<0.05 vs DOX-treated cells.

Then we investigate whether PUN exerts an antioxidative role in DOX-treated H9c2 cells. As demonstrated in Figure 3, increased ROS production was found in the DOX-treated H9c2 cells; however, PUN pretreatment prior to DOX obviously reduced the intracellular ROS accumulation.

### PUN alleviates mitochondrial dysfunction in DOX-treated H9c2 cells

**Figure 4:**
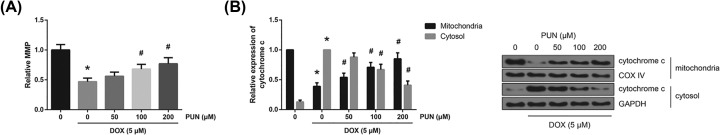
PUN alleviates mitochondrial dysfunction in DOX-treated H9c2 cells (**A**) The loss of MMP in H9c2 cells was determined by JC-1 staining. (**B**) Western blot analysis showed the levels of cytochrome c in the cytoplasm and mitochondria of H9c2 cells. The results are expressed as mean ± S.D. ^*^P<0.05 vs control cells; ^#^*P*<0.05 vs DOX-treated cells.

The mitochondria have been regarded as central executioners of cell death. As shown in Figure 4A, the MMP was remarkably reduced in DOX-stimulated H9c2 cells, and PUN pretreatment prevented the MMP loss. Besides, as indicated by Western blot analysis, DOX stimulation induced the release of mitochondrial cytochrome c into the cytoplasm, and PUN pretreatment concentration-dependently blocked the release (Figure 4B).

### PUN enhances the expression of nuclear Nrf2 in DOX-treated H9c2 cells

**Figure 5:**
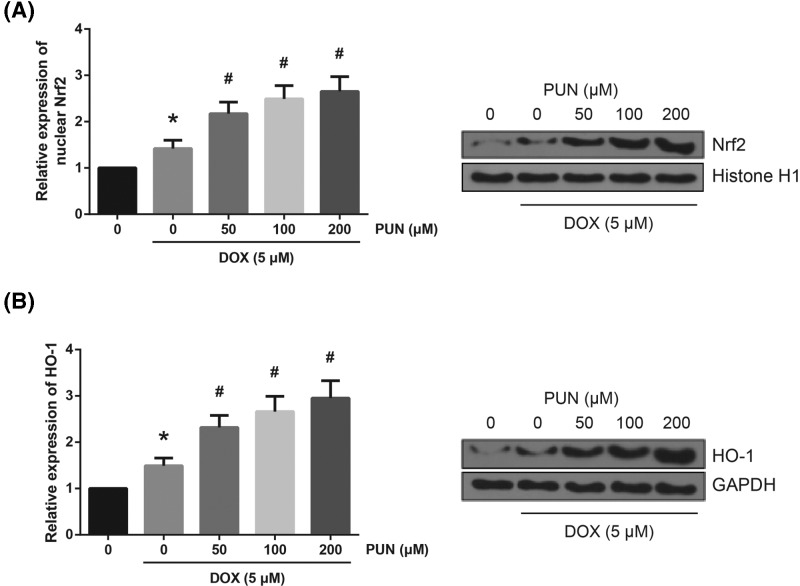
PUN enhances the expression of nuclear Nrf2 in DOX-treated H9c2 cells (**A**) Western blot analysis showed the expression levels of nuclear Nrf2 in H9c2 cells. (**B**) Western blot analysis showed the expression levels of HO-1 in H9c2 cells. The results are expressed as mean ± S.D. ^*^P<0.05 vs control cells; ^#^*P*<0.05 vs DOX-treated cells.

To further explore the mechanisms underlying the effects of PUN, the expression levels of Nrf2 signaling proteins were also analyzed. As shown in Figure 5A, DOX stimulation increased the level of nuclear Nrf2, and PUN pretreatment further enhanced the Nrf2 nuclear translocation. Furthermore, we found that exposure to DOX also increased the protein expression of HO-1, and PUN pretreatment further increased the HO-1 expression (Figure 5B). These data indicated the implication of Nrf2/HO-1 signaling in the protective role of PUN against DOX toxicity.

### Knockdown of Nrf2 abrogates the beneficial role of PUN in DOX-treated H9c2 cells

**Figure 6:**
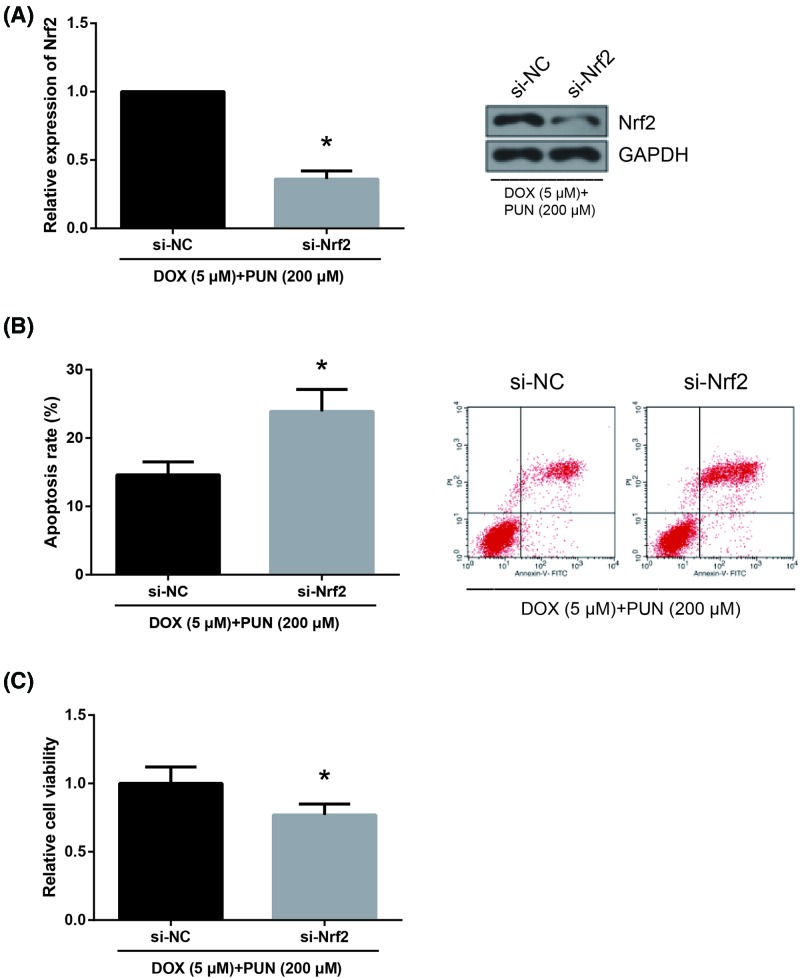
Knockdown of Nrf2 abrogates the beneficial role of PUN in DOX-treated H9c2 cells (**A**) Western blot analysis showed the expression levels of Nrf2 in H9c2 cells. (**B**) The apoptotic rates of H9c2 cells were measured by flow cytometry following Annexin-V/PI staining. (**C**) The cell viability of H9c2 cells was detected by MTT assay. The results are expressed as mean ± S.D. *P<0.05 vs si-NC-transfected cells.

We further clarified the association between Nrf2/HO-1 signaling and the beneficial role of PUN. As confirmed by Western blot analysis, Nrf2 expression was strongly reduced in DOX (5 μM) + PUN (200 μM)-treated H9c2 cells after transfection with si-Nrf2 (Figure 6A). We further observed that transfection with si-Nrf2 obviously blocked the inhibitory effects of PUN on the increased apoptosis and impaired viability of DOX (5 μM)-treated H9c2 cells (Figure 6B,C).

## Discussion

At present, DOX-induced cardiotoxicity remains a major concern in clinical practice, and many therapeutic agents for this serious side-effect were identified. For example, ellagic acid may serve a potent cardiac protective role against DOX [6]. In the present study, we evaluated whether PUN may represent a possible pharmacological intervention in DOX-induced cardiotoxicity. DOX-induced apoptotic death of cardiomyocytes is the most direct cause of DOX-induced cardiotoxicity [7,8], and the present study showed that the impaired viability and increased apoptosis of DOX-stimulated H9c2 cells were rescued by PUN pretreatment.

DOX-induced cardiotoxicity is also attributed to excessive intracellular ROS production [9]. Under pathological circumstances, ROS and oxidative stress may act as the triggers and modulators for cellular apoptosis and death [10]. Cardiomyocytes are rich in mitochondria, a major endogenous source and target of ROS [11]. DOX preferentially accumulates in cardiac mitochondria, and mitochondrial dysfunction is an early indicator of DOX-induced apoptosis [12,13]. As previously reported, stabilization of MMP prevents DOX-induced cardiotoxicity in isolated rat heart [14]. Based on our experimental results, we speculated that PUN decreases ROS generation and improves the mitochondrial function, thereby ultimately reducing the DOX-induced apoptosis of cardiomyocytes.

The transcription factor Nrf2, a major regulator of the adaptive response to exogenous and endogenous oxidative insults [15], is also a critical component in antioxidant defenses in mammalian cardiac cells [16], and Nrf2 deficiency exaggerates DOX-induced cardiotoxicity and cardiac dysfunction [17]. Upon oxidative stress, Nrf2 translocates from cytoplasm into the cell nucleus to induce the expression of HO-1, an inducible antioxidant enzyme [18]. In LPS-stimulated macrophages, PUN induces Nrf2/HO-1 expression and inhibits LPS-induced oxidative stress [19]. The findings of the present study indicated that PUN might help H9c2 cells to fight against DOX-induced oxidative stress through promoting the nuclear translocation of Nrf2 and then elevating HO-1 expression.

In short, for the first time, the present study provides evidence that pretreatment with PUN could attenuate DOX-induced oxidative stress partly through Nrf2/HO-1 signaling. Although further experiments using clinical samples and *in vivo* models are required, our findings indicated that PUN might be a potential therapeutic agent for alleviating DOX-induced cardiotoxicity.

## Abbreviations

DCFH-DA: 2′7′-dichlorodihydrofluorescein diacetate
DOX: doxorubicin
LDH: lactate dehydrogenase
MMP: mitochondrial membrane potential
PUN: punicalagin
S.D.: standard deviation
si-Nrf2: Small interfering RNA targeting rat Nrf2
siRNA: small interfering RNA
