# Expression of Concern: Punicalagin protects H9c2 cardiomyocytes from doxorubicin-induced toxicity through activation of Nrf2/HO-1 signaling

**DOI:** 10.1042/BSR-2019-0229_EOC

**Published:** 2024-11-15

**Keywords:** cardiotoxicity, doxorubicin, mitochondrial dysfunction, Nrf2/HO-1 signaling, punicalagin

The Editorial Office has been made aware of potential issues surrounding the scientific validity of this paper “Punicalagin protects H9c2 cardiomyocytes from doxorubicin-induced toxicity through activation of Nrf2/HO-1 signaling” DOI: 10.1042/BSR20190229, hence has issued an expression of concern to notify readers whilst the Editorial Office investigates. It has been noted that the Western blots and flow cytometry scatter graphs resemble those in other papers by different authors. The authors were contacted regarding the concerns, however so far the Editorial Office has not received a response.

